# Neem oil increases the persistence of the entomopathogenic fungus *Metarhizium anisopliae* for the control of *Aedes aegypti* (Diptera: Culicidae) larvae

**DOI:** 10.1186/s13071-019-3415-x

**Published:** 2019-04-11

**Authors:** Adriano R. Paula, Anderson Ribeiro, Francisco José Alves Lemos, Carlos P. Silva, Richard I. Samuels

**Affiliations:** 1 Department of Entomology and Plant Pathology, Universidade Estadual do Norte Fluminense Darcy Ribeiro, Campos dos Goytacazes, CEP 28013-602 Brazil; 2 Department of Biotechnology, Universidade Estadual do Norte Fluminense Darcy Ribeiro, Campos dos Goytacazes, CEP 28013-602 Brazil; 3 Departamento de Bioquímica, Universidade Federal de Santa Catarina, Florianópolis, 88040-900 Brazil

**Keywords:** Virulence, Adjuvant, Photo-protectant, Bioactive compounds, Fungus, Vector, Dengue, Biological control

## Abstract

**Background:**

The entomopathogenic fungus *Metarhizium anisopliae* is a candidate for the integrated management of the disease vector mosquito *Aedes aegypti*. *Metarhizium anisopliae* is pathogenic and virulent against *Ae. aegypti* larvae; however, its half-life is short without employing adjuvants. Here, we investigated the use of neem oil to increase virulence and persistence of the fungus under laboratory and simulated field conditions.

**Methods:**

Neem was mixed with *M. anisopliae* and added to recipients. Larvae were then placed in recipients at 5-day intervals for up to 50 days. Survival rates were evaluated 7 days after exposing larvae to each treatment. The effect of neem on conidial germination following exposure to ultraviolet radiation was evaluated under laboratory conditions. Statistical tests were carried out using ANOVA and regression analysis.

**Results:**

Laboratory bioassays showed that the fungus alone reduced survival to 30% when larvae were exposed to the treatment as soon as the suspension had been prepared (time zero). A mixture of fungus + neem resulted in 11% survival at time zero. The combination of fungus + neem significantly reduced larval survival rates even when suspensions had been maintained for up to 45 days before adding larvae. For simulated-field experiments 1% neem was used, even though this concentration is insecticidal, resulting in 20% survival at time zero. However, this toxic effect was reduced over time. When used alone under simulated-field conditions the fungus rapidly lost virulence. The formulation fungus + neem effectively maintained fungal virulence, with larval survival rates significantly reduced for up to 45 days after preparation of the suspensions. The effective half-life of the fungus or neem when used separately was 6 and 13 days, respectively. The half-life of fungus formulated in 1% neem was 34 days. Conidia suspended in neem maintained high levels of germination even following a 2-h exposure to ultraviolet radiation.

**Conclusions:**

A combination of the entomopathogenic fungus *M. anisopliae* with neem oil effectively increases the half-life and virulence of the fungus when tested against *Ae. aegypti* larvae, even under simulated field conditions. Neem oil also protected the fungus from the damaging effects of ultraviolet radiation.

**Electronic supplementary material:**

The online version of this article (10.1186/s13071-019-3415-x) contains supplementary material, which is available to authorized users.

## Background

Arboviruses vectored by the mosquito *Aedes aegypti*, namely dengue, Zika, chikungunya and urban yellow fever, are of growing concern, not only in tropical and semi-tropical regions of the world, but also in southern Europe where it is now a threat [1]. Dengue fever is one of the most serious viral diseases vectored by mosquitoes, principally *Ae. aegypti*, affecting 50–100 million people annually, with an estimated 500,000 people requiring hospitalization each year [2]. Vaccines are available for dengue fever and yellow fever; however, the dengue vaccine has certain restrictions [3]. The 2018 yellow fever epidemic in Brazil [4] was probably vectored by mosquitoes of the genus *Haemagogus* and *Sabethes*, which are only found in forested areas, in what is termed the sylvatic cycle [5]. This epidemic resulted in the death of 338 people in 2018. Yellow fever epidemics will become even more serious if the virus enters the urban cycle, where it is normally vectored by *Ae. aegypti*.

Mass vaccinations are underway in Brazil, but to avoid arboviruses spreading rapidly in densely populated urban environments it is necessary to maintain the mosquito populations as low as possible. Conventional control programs are not proving to be effective against *Ae. aegypti*, therefore, alternative approaches are urgently needed.

Conventional control of *Ae. aegypti* normally involves the elimination of breeding sites and chemical insecticide application. One of the main problems associated with chemical control is the rapid development of insecticide resistance, which is particularly serious in Brazil where *Ae. aegypti* larvae have become resistant to the widely used organophosphate temephos [6]. Many populations of adult *Ae. aegypti* are also now resistant to the pyrethroids cypermethrin and deltamethrin [7].

Studies have confirmed the potential of the entomopathogenic fungus *Metarhizium anisopliae* as an alternative for the control of *Ae. aegypti* larvae, by exposing this insect to conidial suspensions in water containers [8, 9]; however, the half-life of the conidia was found to be only ten days under laboratory conditions [8]. Therefore, it would be interesting to increase the persistence of the fungus using appropriate adjuvants. One possible candidate is neem oil.

The advantages of formulating fungi in oil have been well documented in the literature and one of the most important examples is the Lubilosa program (Lutte Biologique contre les Locustes et les Sauteriaux), in which oil formulated conidia were successfully used against the desert locust in Africa [10, 11]. The oil formulation (50:50 Shellsol T:Ondina or 70:30 kerosene:peanut oil) facilitated infection of the locusts even at low humidity (35% RH), normally a limiting factor for fungal germination [10]. Formulating conidia in Shellsol T has also been shown to improve the effectiveness of both *M. anisopliae* and *Beauveria bassiana* against larvae of the malaria mosquitos *Anopheles stephensi* and *Anopheles gambiae* [12]. In that case, the oil aided the dispersal of conidia over the water surface, increasing the chances of the conidia adhering to the larval integument when the mosquitoes came to the surface to breath.

We have recently shown that combining neem oil with *M. anisopliae* significantly increases the virulence of this fungus against *Ae. aegypti* larvae [9], even when this oil was used at low concentrations. Neem oil, when tested on its own, was toxic to *An. stephensi*, *Culex quinquefasciatus* and *Ae. aegypti* larvae [13]. Formulations of entomopathogenic fungi with neem oil have been tested against *An. gambiae* larvae and adults, as well as adult *Cx. quinquefasciatus* [14, 15], with higher mortalities observed when using a combination of fungus + neem than when the agents were tested separately.

Azadirachtin is the main biologically active component of neem oil and the quantity of azadirachtin in neem seeds has been directly correlated with insecticidal activity [16]. Azadirachtin blocks the synthesis and release of developmental hormones such as ecdysteroids, altering the molting process. It also acts as a growth regulator, inhibiting the defense system of the larval cuticle, facilitating the penetration of entomopathogens [17].

Many entomopathogenic fungi are compatible with a range of chemical and natural insecticides [18, 19]. However, antagonistic interactions cannot be discounted without compatibility testing. In the case of neem oil, a study has shown that this compound can inhibit *B. bassiana* vegetative growth, decreasing conidial production and viability [20]. Neem oil, when used at a concentration of less than 1% (v/v), caused no negative effects on the fungus *Metarhizium acridum* and the combination of neem oil with this fungus accelerated locust mortality, increasing the efficiency of this biological control agent [21]. Gomes et al. [9] did not observe any deleterious effects on germination or growth of *M. anisopliae*, even when exposed to 1% (v/v) neem oil.

Here we demonstrate for the first time that neem oil increases not only the virulence of the fungus *M. anisopliae* against *Ae. aegypti* larvae, but also the persistence of the fungus under laboratory and field conditions. This is a significant finding when considering the problems of maintaining the viability of fungi against mosquito larvae, due to the short effective half-life of these organisms under field conditions. One of the most serious problems involving the application of microbial biological control agents in the field is damage caused by exposure to ultraviolet (UV) radiation. Here we also demonstrate for the first time that neem oil acts as a UV protectant, maintaining fungal viability.

## Methods

### Maintenance of insect colonies

*Aedes aegypti* larvae were obtained from field-collected mosquito eggs. Eggs were collected using “ovitraps” deployed around the university campus (21°45′S, 41°17′W), placed in shaded areas. Ovitraps were retrieved on a weekly basis and the hardboard strips onto which the eggs had been laid were placed in trays filled with water to stimulate larval hatching. Only F1 larvae were used in experiments. Larvae were maintained in plastic trays (80 larvae per 100 ml) and fed on freshly ground and autoclaved commercial mouse food (Nuvilab, São Paulo, Brazil) (0.05 g per l). Only second- and third-instar larvae were used in the experiments.

### Fungal isolate and preparation of suspensions

The isolate of *M. anisopliae* used here was obtained from the collection at ESALQ (ESALQ818) in Piracicaba (São Paulo), which had been previously demonstrated to have high virulence against *Ae. aegypti* [8, 9]. Fungi were cultured on Sabouraud Dextrose Agar (SDA: dextrose 10 g. peptone 2.5 g, yeast extract 2.5 g, agar 20 g, in 1 l H_2_O) at 27 °C for 15 days before being used in experiments. Stock fungal suspensions were initially prepared in Tween 80 (0.05% v/v in sterile distilled water) and the conidial concentration was determined using a Neubauer hemocytometer. Final conidial concentrations were prepared by serial dilution in tap water.

### Preparation of neem oil

The commercially produced oil “Base Neem” from the company “Base Fertile Nim” (São Paulo, Brazil) with 0.12% (v/v) azadirachtin, the main insecticidal component, was used in the tests. The oil was diluted with tap water to the desired concentration.

### Persistence test protocol under laboratory conditions

The tests were performed under laboratory conditions at approximately 25 °C, 75% RH, 16:8 L:D photoperiod. For these tests, 4 treatments were used: fungus (1 × 10^9^ conidia ml^-1^) + neem (0.01% v/v), fungus alone, neem alone, and controls (water only). Three 100 ml plastic cups were filled with 50 ml of each treatment and for each time point (11 time points), with a total of 33 cups per treatment group. Freshly ground and autoclaved commercial mouse food was added to each cup at a concentration of 0.01 g per 50 ml.

Ten larvae (stage 2 or 3) were placed in each cup immediately after assembly (time zero) for each treatment. The survival of the larvae was monitored over 7 days by visual observation. This process was repeated every 5 days until the last time point on day 50. Therefore, it was possible to evaluate the persistence of the active components maintained for different time periods under laboratory conditions before the addition of *Aedes* larvae. Each test was carried out three times.

### Persistence test protocol under simulated field conditions

Five hundred milliliters of each test suspension/solution [fungus (1 × 10^9^ conidia ml^-1^) + neem (0.01%; 0.1%; 1% v/v), fungus only, neem only, and controls] was placed in 700 ml black rectangular plant pots (Additional file 1: Figure S1). The pots were placed inside a large cage (115 × 60 × 75 cm) constructed with a wooden frame and covered in mosquito netting (Additional file 1: Figure S2) maintained on a covered veranda of the insectary at Universidade Estadual do Norte Fluinense (UENF). This was in order to prevent mosquitoes using the pots as oviposition sites. Freshly ground and autoclaved commercial mouse food was added to each pot at a concentration of 0.02 g per 500 ml.

The pots with the different suspensions/solutions were all prepared at the same time but the larvae were only exposed to the treatments at 5-day intervals. For example, at time zero, larvae were added to freshly prepared suspensions/solutions. On day 5, larvae were exposed to treatments which had been maintained under simulated field conditions for 5 days. Subsequently, larvae were then added to the pots at 5-day intervals until the last time point on day 50. The survival of the larvae was monitored by visual observation for 7 days after addition to the pots. Each test was carried out three times.

### Germination rates of conidia following exposure to UV

These experiments were carried out under laboratory conditions with the aim of investigating the possibility that neem oil protects conidia from the deleterious effects of UV radiation. Conidia (1 × 10^8^ conidia ml^-1^) were suspended in Tween 80 (TW 80) (0.05% v/v) and subsequently neem oil was added to a final concentration of 1% and 0.01% (v/v). Fungal suspensions were also prepared in TW 80 without neem. Three milliliters of each sample was placed in plastic Petri dishes (55 mm diameter) and exposed to a UV-B lamp (Osram; Ultra-Vitalux^®^ 300 W), which at a distance of 70 cm was equivalent to a UV-B intensity of 1630 mW m^-2^. Conidia were exposed to UV radiation for 0.5, 1, 2, 3, 4 and 5 h periods. Controls for each treatment were not exposed to UV light.

Conidial germination rates were evaluated by inoculating 15 μl of each suspension onto solid culture media (SDA) and incubating at 27 °C (12 h light: 12 h dark regime) for 12 h. At this time the number of conidia germinating was quantified using an optical microscope (FWL 1000; Feldmann Wild Leitz, São Paulo, Brazil). Three fields of view were counted for each sample by the same researcher (AR). Conidia were considered as germinated when the germ tube was of equal size or larger than the conidia itself. Each treatment was replicated three times.

### Statistical analysis

Mean survival rates were compared using one-way analysis of variance. When significant differences were seen between treatments or between time points within the same treatment, the data was further analyzed by Duncan’s post hoc test at the 5% level. The persistence/half-life of the different treatments was evaluated by using polynomial regression analysis, with the effective half-life given as the time (in days) that the treatments resulted in 50% larval survival/mortality. Differences in mean germination rates following exposure to UV were compared using one-way analysis of variance. When significant differences were detected, Duncan’s *post-hoc* test at the 5% level was used.

## Results

### Fungal persistence under laboratory conditions

Before testing fungal persistence in the field, bioassays were carried out under laboratory conditions using 0.01% neem (v/v), fungus (1 × 10^9^ conidia ml^-1^) and a combination of neem + fungus, with larvae exposed to the different treatments immediately following preparation (time zero) or following different time periods of up to 50 days. The results for these experiments are shown in Table 1. In fact, only the treatment with fungus + neem lasted 50 days, whilst evaluations of the other treatments were stopped when the survival rates were no longer statistically different to the controls. The survival rate of *Ae. aegypti* larvae when exposed to the fungus alone was 30% at time zero (larvae immediately added to the recipients with freshly prepared fungal suspensions), whilst for neem alone the larval survival rate was 74% at time zero. However, exposure of larvae to the formulation fungus + neem resulted in 11% survival at time zero. Fungus + neem significantly reduced survival rates when compared to the other two treatments and to the controls (*F*_(3,11)_ = 1446.2, *P* < 0.01). The survival rates for larvae exposed to the fungus alone were not significantly different (*P* > 0.01) to the controls following 10 days under laboratory conditions, showing the low half-life of the fungus when suspended in TW 80. The slight but significant reduction in survival seen at time zero for neem alone was transitory, with no significant reduction in survival by day 5 (81%) when compared to controls (83%).

**Table 1:** Survival rates of *Aedes aegypti* larvae following exposure to different treatments under laboratory conditions

Time (days)	Survival rate (%)			
	F + N	F	N	Control
0	11 ± 10^Aa^	30 ± 8^Ab^	74 ± 3^Ac^	88 ± 2^Ad^
5	13 ± 9^Ba^	52 ± 5^Bb^	81 ± 3^Bc^	83 ± 2^ABc^
10	17 ± 9^Ca^	84 ± 2^Cb^	–	81 ± 2^ABb^
15	22 ± 8^Ca^	–	–	80 ± 2^ABb^
20	27 ± 9^Da^	–	–	78 ± 2^Bb^
25	30 ± 7^Da^	–	–	84 ± 2^ABb^
30	33 ± 8^Ea^	–	–	81 ± 2^ABb^
35	40 ± 8^Fa^	–	–	83 ± 1^ABb^
40	48 ± 7^Ga^	–	–	78 ± 3^Bb^
45	58 ± 4^Ha^	–	–	77 ± 2^Bb^
50	74 ± 3^Ia^	–	–	83 ± 1^ABa^

*Notes*: The different suspensions/solutions were all prepared at the same time but the larvae were only added to the treatments at 5-day intervals. Time zero: larvae added to freshly prepared suspensions/solutions. Different capital letters show statistical differences within each treatment over time (columns) at the 5% level using Duncan’s post hoc test. Different lower case letters show statistical differences between treatments for the same time point (lines) at the 5% level

*Abbreviations*: F, *Metarhizium anisopliae* conidial suspension (1 × 10^9^ conidia ml^-1^); N, neem oil 0.01%

The survival rates were then monitored for the formulation fungus + neem maintained in recipients for different periods of up to 50 days. The mixture of fungus + neem continued to significantly reduce larval survival rates when maintained in recipients for up to 45 days, with 58% survival observed at this time (*F*_(21,65)_ = 157.5, *P* < 0.01). When larvae were exposed to this formulation following 50 days in recipients, the survival rate was 74%, which was not significantly different to the controls (*P* > 0.01). The half-life of this formulation under laboratory conditions was 42.8 days as calculated by regression analysis (Y = -0.02x^2^ - 0.062x + 85.3; *R*^2^ = 0.98).

### Fungal persistence under field conditions

These tests were similar to those described above but with recipients maintained under field conditions. Two preliminary experiments were carried out but both were stopped after 10 days. The first experiment was set up using the same concentration of neem as that used in laboratory experiments, 0.01% (v/v). However, none of the treatments were significantly different to the controls (*P* > 0.01) following 10 days of maintaining the fungus or neem oil under field conditions (Additional file 1: Table S1). Initially (time zero), both fungus + neem and fungus alone reduced larval survival to 26 and 28%, respectively, significantly different to neem alone (70%) or the controls (*F*_(3,11)_ = 236.9, *P* < 0.01), but larvicidal activity was rapidly lost. Therefore, the next set of experiments was carried out using 0.1% (v/v) neem.

However, a similar pattern was seen for the experiments using 0.1% neem, with larvicidal activity rapidly reduced after 10 days under field conditions (Additional file 1: Table S2). Although the results at 10 days for fungus + neem (71% survival) and fungus alone (72% survival) were statistically different to neem alone and to the controls (*F*_(3,11)_ = 441.0, *P* < 0.01), the experiment was stopped due to the rapid decline in larvicidal activity.

**Fig. 1:**
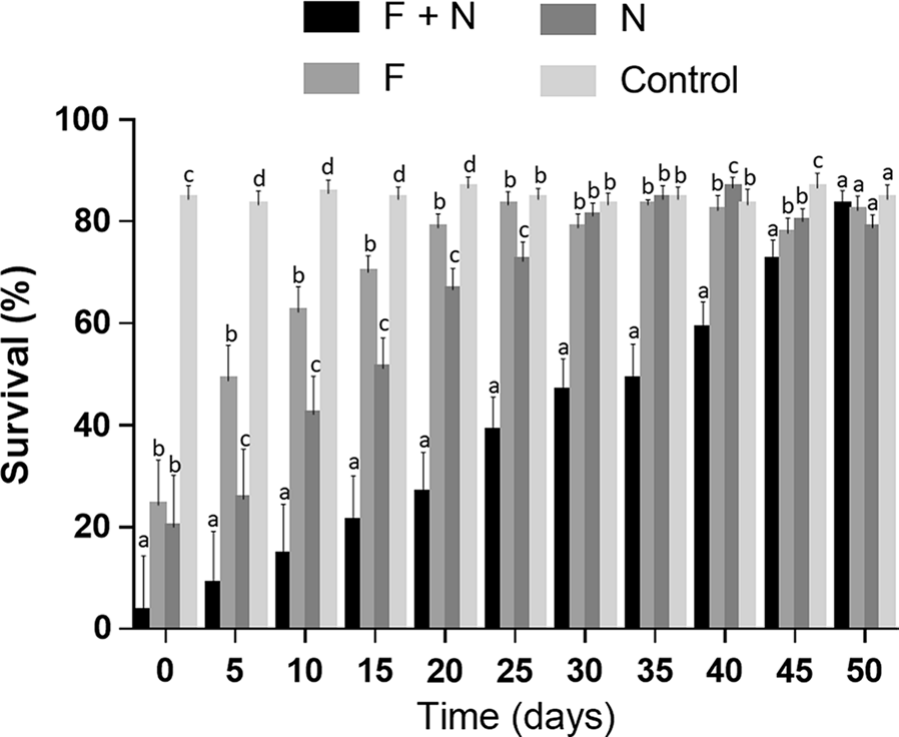
Survival rates of *Aedes aegypti* larvae following exposure to different treatments maintained under simulated field conditions for up to 50 days. The different suspensions/solutions were all prepared at the same time and larvae added to the pots at 5-day intervals. Time zero: larvae added to freshly prepared suspensions/solutions. Different letters show statistical differences (5% level) when comparing survival rates for each treatment at each time point using Duncan’s post hoc test. *Abbreviations*: F, *Metarhizium anisopliae* conidial suspensions (1 × 10^9^ conidia ml^-1^); N, neem oil 1% (v/v)

The next set of field experiments were performed using 1% (v/v) neem (Fig. 1). The results for the persistence of 1% (v/v) neem + fungus were very promising. This formulation was highly effective at reducing larval survival at time zero, with only 3% of the larvae remaining alive after exposure to 1% (v/v) neem + fungus seven days after exposing the larvae to this treatment. This result was significantly different to the two other treatments and the controls (*F*_(3,11)_ = 478.4, *P* < 0.01). At time zero, the fungus alone significantly reduced larval survival (24% survival) when compared to the controls, as did neem alone (20% survival), with no significant difference between these two treatments (Fig. 1).

The persistence of the formulations was evaluated for up to 50 days under simulated field conditions. When comparing survival rates of all treatments with controls over time, the formulation fungus + neem maintained significant larvicidal activity until day 45 (72% survival), at which time the survival rates were statistically different to the other treatments and the controls (*F*_(21,65)_ = 556.6, *P* < 0.01). Fifty days after maintaining this formulation under simulated field conditions, and then adding larvae to the recipients, fungus + neem was no longer effective at reducing survival rates and there was no significant difference between the treatments.

The fungus when tested alone under field conditions rapidly lost virulence, with no difference to the controls at 25 days, when survival rates were 83% (*P* > 0.01). Neem alone caused significant reductions in survival (72%) until day 25 when compared to the controls.

**Fig. 2:**
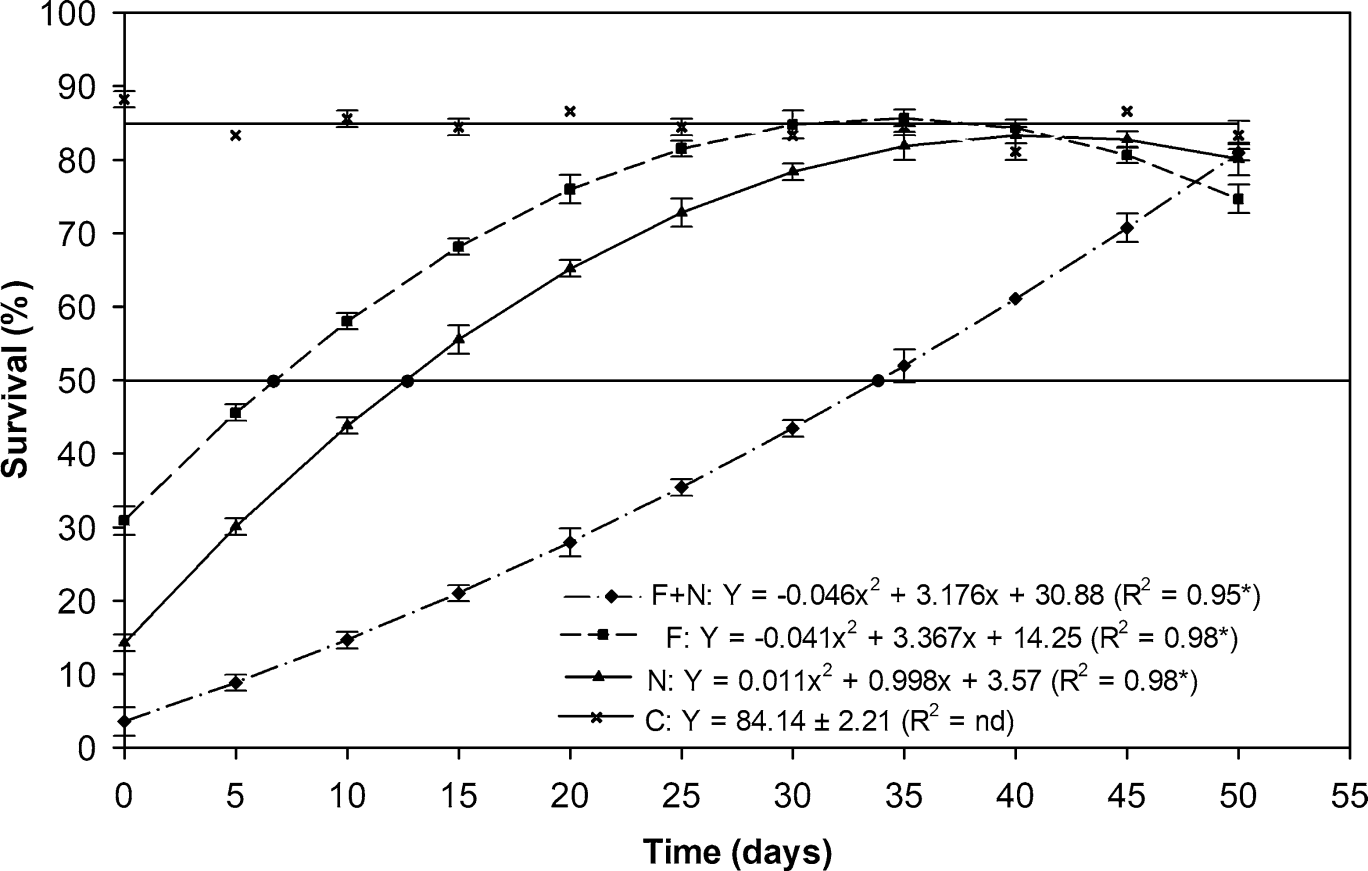
Effective half-life of three treatments used under field conditions as estimated from larval survival rates. Regression analysis was used to calculate the effective half-life of the three treatments: fungus + neem oil (F + N); fungus alone (F); and neem oil alone (N). The horizontal line at 50% survival, which bisects the regression curves, was used to estimate half-life. The precise half-life was calculated by regression analysis and the values for Y and *R*^2^ and shown (insert)

Using regression analysis is was possible to estimate the effective half-life of these treatments; in other words, the amount of time the agents could be maintained in the field before adding *Ae. aegypti* larvae and still result in a 50% reduction in survival. Figure 2 shows the regression curves for all treatments. The *R*^2^ value for neem + fungus was 0.95 and the half-life was 34 days. The results showed that the half-life of the fungus alone or neem alone was only 6 and 13 days, respectively.

### Germination rates of conidia following exposure to UV

**Fig. 3:**
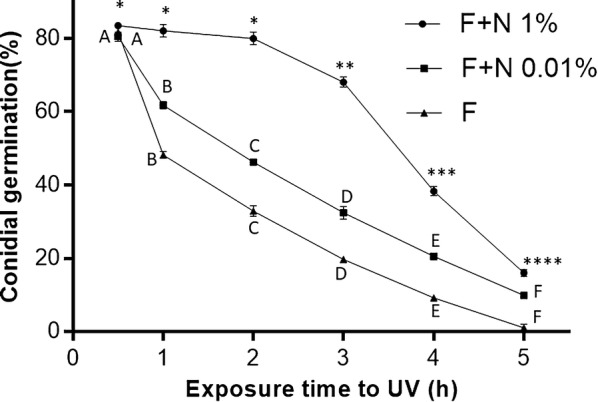
Rate of conidial germination following different exposure periods to ultraviolet light. Conidia were exposed to UV-B light for different periods of up to 5 h, either suspended in Tween alone (F) or in two different concentrations of neem oil (F + N: 0.01% or 1% v/v). The results are shown as mean germination rates ± SD (bars). Different letters or symbols indicate statistically different germination rates when comparing within treatment results

The results for the three treatments investigated here showed the significant protective characteristics of neem when considering the deleterious effects of UV radiation on conidial germination (Fig. 3). When conidial suspensions were exposed to UV without neem, the germination rate was rapidly reduced, with only 48% germination observed after exposing conidial suspension to UV-B radiation for 1 h. In the case of conidial suspensions formulated with neem oil, 1% (v/v) neem provided greater protection than 0.01% (v/v) neem. No significant reductions in germination rates were seen following a 2 h exposure of UV when conidia were suspended in 1% (v/v) neem oil (*F*_(5,17)_ = 46.07, *P* < 0.01), with 80.3% (± 1.69%) germination observed. However, 3 to 5 h exposures caused significant reductions in germination when compared to shorter exposure times. Neem 0.01% (v/v) was a less effective protectant, with rapid and significant reductions in conidial germination when exposing conidia to UV-B for 1 h or more (*F*_(5,17)_ = 9270.0, *P* < 0.01). All treatments showed low levels of germination following a 5-h exposure to UV. The mean germination rates of all three control treatments were not significantly different (*P* > 0.01) and approximately 80% of the conidia had germinated when quantified 12 h after inoculating the suspensions in culture media. The complete data and statistical analysis for the germination experiments are shown in Additional file 1: Table S3.

## Discussion

Current conventional mosquito larval control measures in domestic environments involves local government agents visiting residences and treating putative breeding sites, such as drains and water tanks with synthetic chemical larvicides or products containing *Bacillus thuringiensis* serotype *israelensis* (Bti). When Bti is applied in the field in recipients exposed to sunlight, control is efficient for up to four weeks. However, when used in recipients not directly exposed to sunlight, the product is effective for up to 11 weeks [22].

One of the most serious drawbacks of deploying entomopathogenic fungi for pest and vector control is their short effective half-life under field conditions. With the aim of increasing the persistence of these fungi, many studies have been carried out on different formulation technologies [23]. Amongst these, a range of oil-based formulations have been utilized as adjuvants of entomopathogenic fungi, the most well-known example being the mixture of dry *Metarhizium* conidia in mineral oil in a product known as Green Muscle^®^, used for the control of the desert locust [11]. In this case, the oil-based formulation permitted the use of fungi under field conditions with low humidity, the oil forming a microclimate suitable for increased fungal persistence on the insect integument and on plant surfaces. This formulation also increased the frequency of secondary infection of locusts that came into contact with surfaces on to which conidia had been sprayed [10].

In our previous study of neem oil as an adjuvant for use with entomopathogenic fungi, very low concentrations of neem oil significantly increased the virulence of *M. anisopliae* against *Ae. aegypti* larvae [9], probably as a result of increasing stress and reducing immune responses due to low level intoxication. In the present study, neem oil has not only been confirmed to increase virulence but also to increase the persistence of the fungus under laboratory and field conditions.

Neem oil has insecticidal properties and is currently used for pest control in organic farming systems [24]. However, it is relatively expensive for deployment on a large scale. Neem oil is toxic to a range of mosquitoes [13, 14, 16]. For example, when *Ae. aegypti* larvae were exposed to neem oil, their development was affected, resulting in a significant reduction in adult emergence [13, 25]. Feeding behavior and oviposition rates of the malaria vectors *An. stephensi* and *An. gambiae* were reduced following exposure to neem. Pupal formation and adult emergence were significantly reduced in the case of *An. stephensi* and *An. gambiae* larvae exposed to neem [26, 27].

In the first set of experiments here, under laboratory conditions, the concentration of neem that increased fungal persistence was not considered to be toxic to *Ae. aegypti* larvae. Neem when used at 0.01% (v/v) together with *M. anisopliae* enabled the fungus to significantly reduce larval survival rates for up to 45 days after the suspension had been prepared and maintained on the bench. The fungus when used alone rapidly lost virulence, with survival rates equal to that of the controls after only 10 days. The decline in virulence of *M. anisopliae* seen here was greater than that seen previously, when the half-life of the same fungus was estimated to be 10 days under similar conditions [8].

The persistence of the fungus when formulated with neem oil was tested under field conditions. The present field experiments were performed using black plant pots, which are highly suitable as *Ae. aegypti* oviposition sites. The same type of plant pot is normally used as a so called “ovitrap” to collect eggs in the field. Although in the present experiments, mosquito larvae were added to the pots to test the persistence of the formulations, future experiments will be carried out using ovitraps with fungus + neem in the field as a possible control strategy against the egg stage and any larvae emerging from the eggs. However, we have yet to test if this formulation could be detected by females and act as a repellant to oviposition. Studies have shown that *Ae. aegypti* females are not repelled by the presence of *M. anisopliae* conidia on black cloths [28]; however, this does not preclude the possibility that females may avoid oviposition sites due to the presence of fungal propagules in the water. It is interesting to note that “beneficial symbiotic microbes” in the water can attract female *An. gambiae*, which oviposit higher numbers of eggs in the presence of bacteria than in sterile water [29].

The initial results for field tests (Additional file 1: Tables S1–S2) showed that the concentrations of neem required under these more rigorous conditions were higher than those required under laboratory conditions. The concentration of neem which significantly increased conidial half-life in the lab (0.01% v/v) was ineffective in the field, as was 0.1% (v/v) neem. Tests were thus performed using 1% neem, although this concentration was toxic to *Ae. aegypti* larvae. Neem at 1% (v/v) has been previously shown to cause a 50% reduction in survival rates within two days [9]. Daily survival rates were not evaluated here, but larval exposure to 1% (v/v) neem resulted in a 20% survival rate after a seven-day exposure to a recently prepared solution of neem oil, in other words, 80% mortality. Neem oil, when used at a 5% (v/v) concentration, killed 95% of *An. stephensi* larvae [16]. Although 1% (v/v) neem was toxic to *Ae. aegypti* larvae when used alone, the advantage of formulating *M. anisopliae* with this concentration of neem was a significant increase in the fungal half-life in the field. The half-life of the fungus alone was only six days, which would seriously impair its use under field conditions. The larvicidal activity of neem also fell rapidly under these conditions, with an estimated half-life of 13 days seen here. This was probably due to bio-degradation.

One of the most important abiotic factors negatively influencing the persistence of entomopathogenic fungi in the field is UV radiation. Both UV-A and UV-B radiation have been shown to reduce conidial germination rates [30]. However, when considering the effects of UV radiation on fungi in an aquatic habitat, water can protect *M. anisopliae* conidia from UV-B [31]. Successful control of the desert locust *Schistocerca gregaria* has been attributed to the use of *Metarhizium* in oil formulations that increased fungal persistence and eliminated the negative effects of low humidity [32].

No studies to date have shown the possible protective properties of oil formulations in an aquatic habitat. However, a recent study has shown that formulating *B. bassiana* conidia in sesame or colza oil increases the fungal persistence when applied to leaf surfaces, protecting the conidia from the damaging effects of UV-B [33].

In order to investigate the possible UV protectant properties of neem oil, we exposed conidial suspensions with and without neem to UV-B radiation. For the control treatments (no neem oil), exposure of conidia to UV rapidly reduced viability. However, conidial suspensions in 1% neem oil protected the conidia for up to two hours exposure to UV, with no significant reductions in germination when compared to controls. For sesame or colza oil, the protective capacity was approximately 70% following a five-hour exposure to UV-B following field trails [33].

## Conclusions

Neem was tested in the laboratory and under field conditions as an adjuvant for the entomopathogenic fungus *M. anisopliae*. This work shows for the first time an important strategy for maintaining fungal virulence over extended time periods. One of the major drawbacks of using entomopathogenic fungi is their short half-life. However, the addition of neem to conidial suspensions not only increased virulence, but significantly increased the persistence of the fungus.

## Additional file

**Additional file 1: Figure S1.** Black rectangular plant pot used for field testing the virulence and persistence of the fungal and neem oil treatments against *Ae. aegypti* larvae. **Figure S2.** Field testing the persistence of the conidia against *Ae. aegypti* larvae. Experiments on a covered veranda were carried out within netting cages to prevent mosquitoes using the plant pots as oviposition sites. **Table S1.** Survival rates of *Ae. aegypti* larvae following exposure to different treatments under simulated field conditions. **Table S2.** Survival rates of *Ae. aegypti* larvae following exposure to different treatments under simulated field conditions. **Table S3.** Germination rates (mean % ± SD) of *Metarhizium anisopliae* conidia following exposure to UV-B when formulated in two concentration of neem oil or Tween.

## Abbreviations

ANOVA: analysis of variance
RH: relative humidity
UV: ultra-violet radiation
TW 80: Tween 80
l:d: light:dark regime
v/v: volume/volume

## References

[1] Reiter P (2010). Yellow fever and dengue: a threat to Europe?. Euro Surveill.

[2] World Health Organization. Dengue and severe dengue. 2017. http://www.who.int/mediacentre/factsheets/fs117/en/. Accessed 08 Nov 2017.

[3] Ferguson NM, Rodriguez-Barraquer I, Dorigatti I, Mier-Y-Teranromero L, Laydon DJ, Cummings DAT (2016). Benefits and risks of the Sanofi-Pasteur dengue vaccine: modeling optimal deployment. Science.

[4] Ministério da Saúde do Brasil. Febre amarela: Ministério da Saúde atualiza casos no país. 2018. http://portalms.saude.gov.br/noticias/agencia-saude/42655-febre-amarela-ministerio-da-saude-atualiza-casos-no-pais. Accessed 18 Apr 2018.

[5] Barrett ADT, Higgs S (2007). Yellow fever: a disease that has yet to be conquered. Ann Rev Entomol..

[6] Montella IR, Martins AJ, Viana-Medeiros PF, Lima JB, Braga IA, Valle D (2007). Insecticide resistance mechanisms of Brazilian *Aedes aegypti* populations from 2001 to 2004. Am J Trop Med Hyg.

[7] da-Cunha MP, Lima JB, Brogdon WG, Moya GE, Valle D (2005). Monitoring of resistance to the pyrethroid cypermethrin in Brazilian *Aedes aegypti* (Diptera: Culicidae) populations collected between 2001 and 2003. Mem Inst Oswaldo Cruz..

[8] Pereira CR, Paula AR, Gomes SA, Pedra PCO, Samuels RI (2009). The potential of *Metarhizium anisopliae* and *Beauveria bassiana* isolates for the control of *Aedes aegypti* (Diptera: Culicidae) larvae. Biocontrol Sci Technol.

[9] Gomes SA, Paula AR, Ribeiro A, Paula CO, Santos JÁ, Silva CP, Samuels RI (2015). Neem oil increases the efficiency of the entomopathogenic fungus *Metarhizium anisopliae* for the control of *Aedes aegypti* (Diptera: Culicidae) larvae. Parasit Vectors.

[10] Bateman RP, Carey M, Moore D, Prior C (1993). The enhanced infectivity of *Metarhizium flavoviride* in oil formulations to desert locusts at low humidities. Ann Appl Biol..

[11] Bateman R, Jenkins N, Kooyman C, Moore D, Prior C, Lacey LA (2017). LUBILOSA: the development of an acridid-specific mycoinsecticide. Microbial control of insect and mite pests, chapter 23.

[12] Bukhari T, Takken W, Koenraadt CJM (2011). Development of *Metarhizium anisopliae* and *Beauveria bassiana* formulations for control of malaria mosquito larvae. Parasit Vectors..

[13] Dua VK, Pandey AC, Raghavendra K, Gupta A, Sharma T, Dash AP (2009). Larvicidal activity of neem oil (*Azadirachta indica*) formulation against mosquitoes. Malar J..

[14] Seye F, Ndiaye M, Faye O, Afoutou JM (2012). Evaluation of entomopathogenic fungus *Metarhizium anisopliae* formulated with suneem (neem oil) against *Anopheles gambiae s.l.* and *Culex quinquefasciatus* adults. Malar Chemother Cont Elim.

[15] Seye F, Ndione D, Touré M, Ndiaye M, Boukraa S, Bawin T (2013). Laboratory and semi-field environment tests for the control efficacy of *Metarhizium anisopliae* formulated with neem oil (suneem) against *Anopheles gambiae s.l.* adult emergence. Acad J Biotechnol.

[16] Murugan K, Babu R, Jeyabalan D, Kumar NS, Sivaramakrishnan S (1996). Antipupational effect of neem oil and neem seed kernel extract against mosquito larvae of *Anopheles stephensi* (Liston). J Entomol Res.

[17] Mulla MS, Su T (1999). Activity and biological effects of neem products against arthropods of medical and veterinary importance. J Am Mosq Control Assoc.

[18] Santos A, Oliveira BL, Samuels RI (2007). Selection of entomopathogenic fungi for use in combination with sub-lethal doses of imidacloprid. Mycopathology..

[19] Pelizza SA, Scorsetti AC, Fogel MN, Pacheco-Marino SG, Stenglein SA, Cabello MN (2015). Compatibility between entomopathogenic fungi and biorational insecticides in toxicity against *Ronderosia bergi* under laboratory conditions. Biocontrol.

[20] Depieri RA, Martinez SS, Menezes JRAO (2005). Compatibility of the fungus *Beauveria bassiana* (Bals.) Vuill. (Deuteromycetes) with extracts of neem seeds and leaves and the emulsible oil. Neotrop Entomol.

[21] Haroona WM, Pagesb C, Vassalb J-M, Abdallaa AM, Luong-Skovmandb M, Lecoq M (2011). Laboratory and field investigation of a mixture of *Metarhizium acridum* and neem seed oil against the tree locust *Anacridium melanorhodon melanorhodon* (Orthoptera: Acrididae). Biocontrol Sci Technol.

[22] Vilarinhos PTR, Monnerat R (2004). Larvicidal persistence of formulations of *Bacillus thuringiensis* var. *israelensis* to control larval *Aedes aegypti*. J Am Mosq Control Assoc..

[23] Fernandes ÉKK, Rangel DEN, Braga GUL, Roberts DW (2015). Tolerance of entomopathogenic fungi to ultraviolet radiation: a review on screening of strains and their formulation. Curr Genet.

[24] Campos EVR, de Oliveira JL, Pascoli M, de Lima R, Fraceto LF (2016). Neem oil and crop protection: from now to the future. Front Plant Sci..

[25] Ndione RD, Faye O, Ndiaye M, Dieye A, Marie J (2007). Toxic effects of neem products (*Azadirachta indica* A. Juss) on *Aedes aegypti* Linnaeus, 1762 larvae. J Biotechnol.

[26] Nathan SS, Kalaivani K, Murugan K (2005). Effects of neem limonoids on the malaria vector *Anopheles stephensi* Liston (Diptera: Culicidae). Acta Trop.

[27] Okumu FO, Knols BGJ, Fillinger U (2007). Larvicidal effects of a neem (*Azadirachta indica*) oil formulation on the malaria vector *Anopheles gambiae*. Malar J.

[28] Paula AR, Carolino AT, Silva CP, Pereira CR, Samuels RI (2013). Testing fungus impregnated cloths for the control of adult *Aedes aegypti* under natural conditions. Parasit Vectors..

[29] Sumba LA, Guda TO, Deng AL, Hassanali A, Beier JC, Knols BGJ (2004). Mediation of oviposition site selection in the African malaria mosquito *Anopheles gambiae* (Diptera: Culicidae) by semiochemicals of microbial origin. Int J Trop Insect Sci.

[30] Braga GUL, Flint SD, Miller CD, Anderson AJ, Roberts DW (2001). Both solar UVA and UVB radiation impair conidial culturability and delay germination in the entomopathogenic fungus *Metarhizium anisopliae*. Photochem Photobiol.

[31] Falvo ML, Albornoz Medina P, Rodrigues J, Lopez Lastra CC, García JJ, Fernandes EKK, Luz C (2018). Effect of UV-B irradiation on water-suspended *Metarhizium anisopliae s.l.* (Hypocreales: Clavicipitaceae) conidia and their larvicidal activity in *Aedes aegypti* (Diptera: Culicidae). J Med Entomol..

[32] Lomer CJ, Bateman RP, Johnson DL, Langewald J, Thomas M (2001). Biological control of locusts and grasshoppers. Annu Rev Entomol.

[33] Kaiser D, Bacher S, Mène-Saffrané L, Grabenweger G (2019). Efficiency of natural substances to protect *Beauveria bassiana* conidia from UV radiation. Pest Manag Sci.

