# Supplementary material for: Neem oil increases the persistence of the entomopathogenic fungus Metarhizium anisopliae for the control of Aedes aegypti (Diptera: Culicidae) larvae
Source: Parasit Vectors. 2019 Apr 11;12:163. doi: 10.1186/s13071-019-3415-x (PMC6460681; doi:10.1186/s13071-019-3415-x)
Supplement: Supplementary file 1 — Additional file 1: Figure S1. Black rectangular plant pot used for field testing the virulence and persistence of the fungal and neem oil treatments against Ae. aegypti larvae. Figure S2. Field testing the persistence of the conidia against Ae. aegypti larvae. Experiments on a covered veranda were carried out within netting cages to prevent mosquitoes using the plant pots as oviposition sites. Table S1. Survival rates of Ae. aegypti larvae following exposure to different treatments under simulated field conditions. Table S2. Survival rates of Ae. aegypti larvae following exposure to different treatments under simulated field conditions. Table S3. Germination rates (mean % ± SD) of Metarhizium anisopliae conidia following exposure to UV-B when formulated in two concentration of neem oil or Tween. [file 13071_2019_3415_MOESM1_ESM.docx]

**Additional file 1**


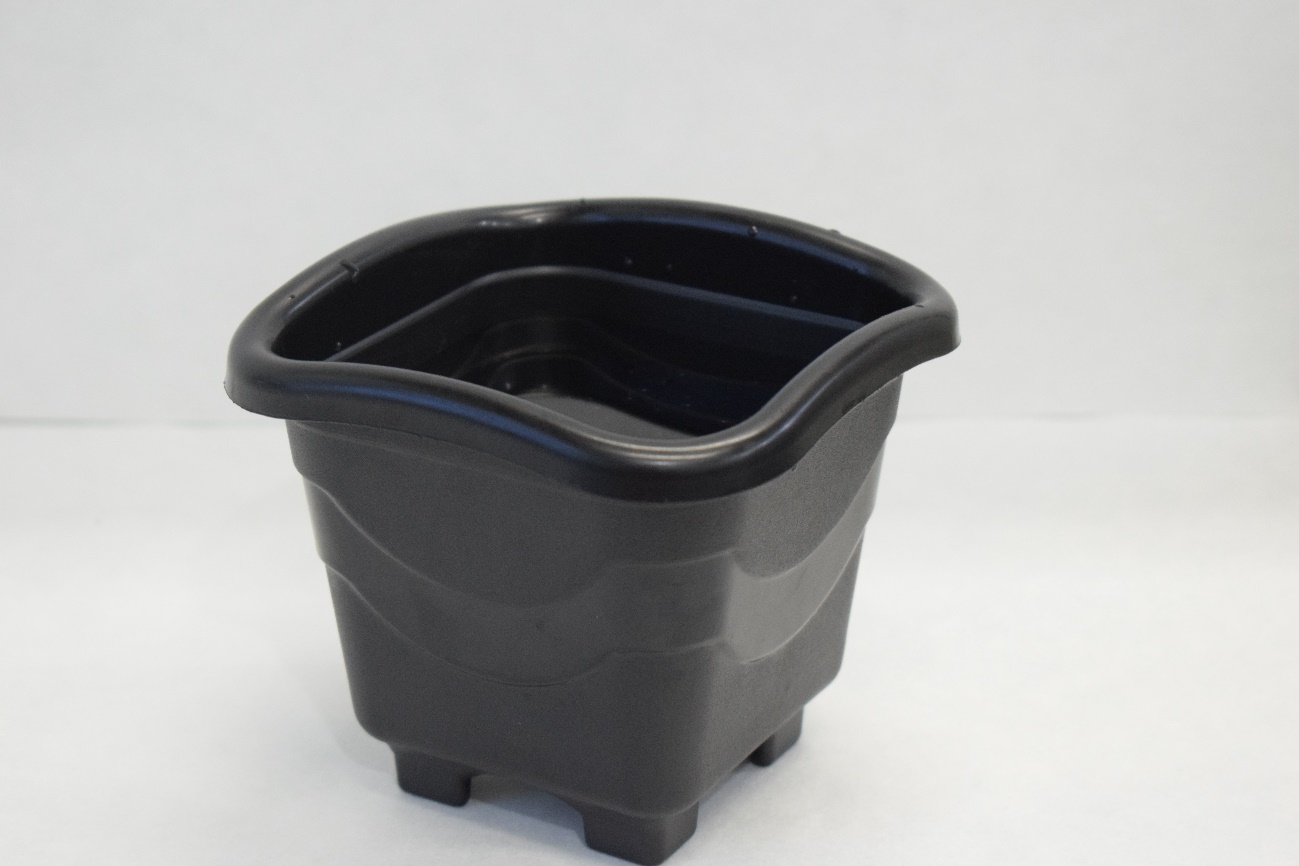


**Figure S1.** Black rectangular plant pot used for field testing the virulence and persistence of the fungal and neem oil treatments against *Aedes aegypti* larvae.


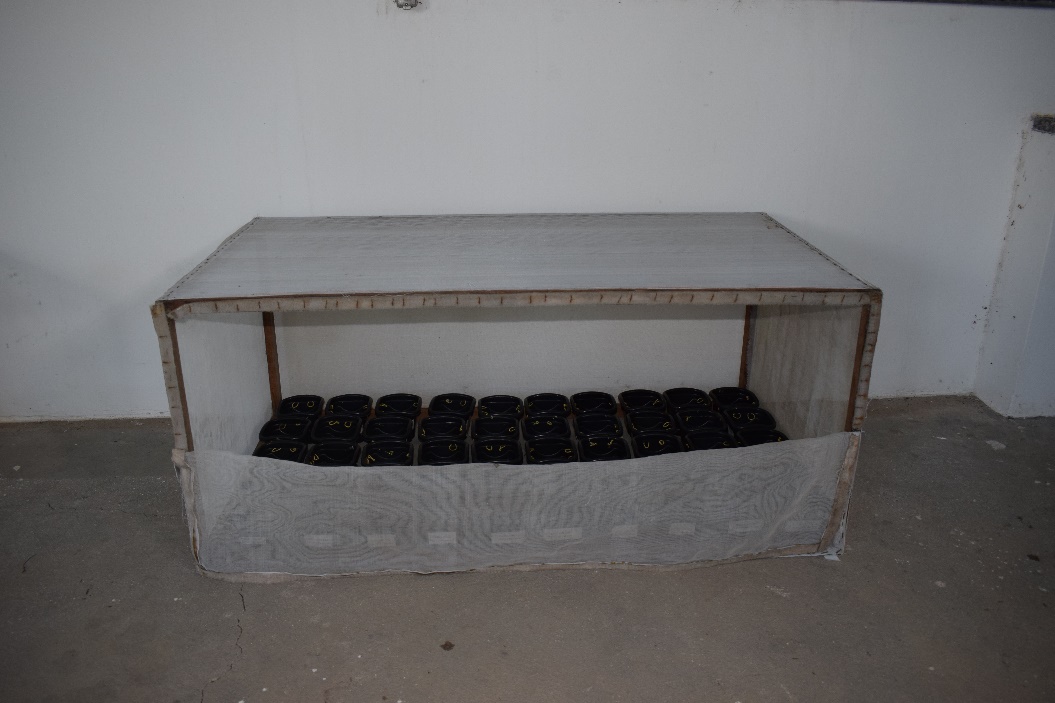


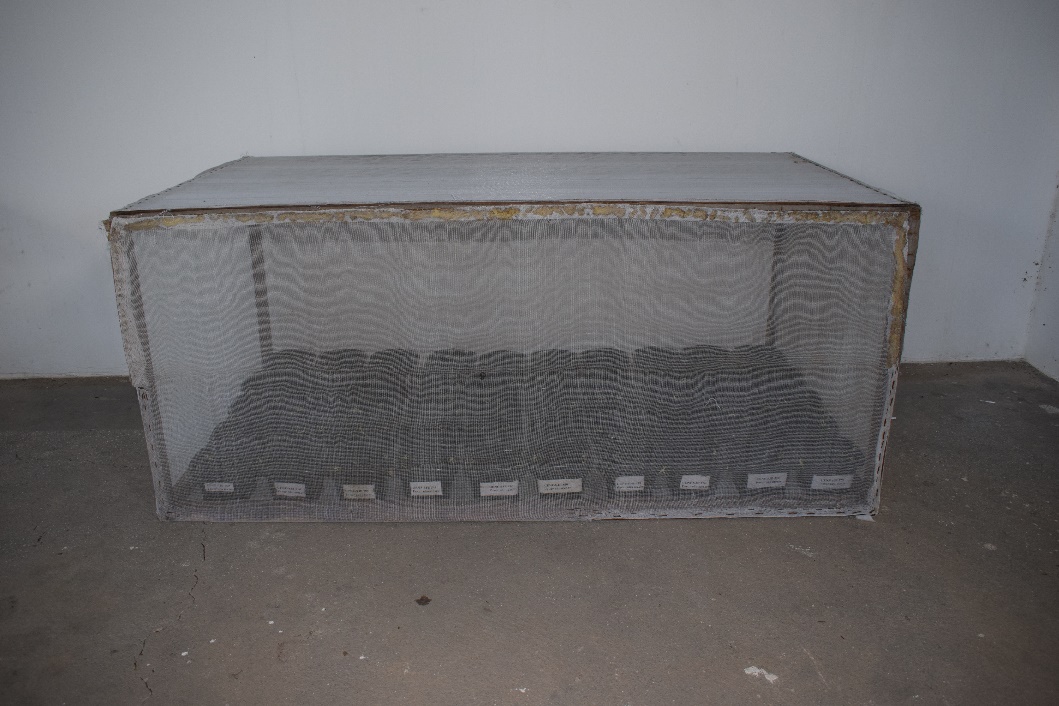


**Figure S2** Field testing the persistence of the conidia against *Aedes aegypti* larvae.

Experiments on a covered veranda were carried out within netting cages to prevent mosquitoes using the plant pots as oviposition sites.

**Table S1 Survival rates of *Aedes aegypti* larvae following exposure to different treatments under simulated field conditions**

| **SURVIVAL RATES (%)** | | | | |
| --- | --- | --- | --- | --- |
| **TIME (days)** | **F + N** | **F** | **N** | **CONTROLS** |
| 0 | 26.6 ± 8.6 Aa | 28.8 ± 7.3 Aa | 70 ± 4.0 Ab | 82.2 ± 2.6 Ac |
| 5 | 48.8 ± 6.3 Ba | 52.2 ± 5.2 Ba | 83.3 ± 3.3 Bb | 83.3 ± 2.4 Ab |
| 10 | 83.3 ± 2.9 Ca | 82.2 ± 2.6 Ca | 85.5 ± 3.2 Ba | 84.4 ± 2.9 Aa |

**F:** *Metarhizium anisopliae* conidial suspensions (1x10^9^ conidia mL^-1^)

**N**: Neem oil 0.01%

The different suspensions/solutions were all prepared at the same time but the larvae were only added to the treatments at 5-day intervals. Time zero: larvae added to freshly prepared suspensions/solutions.

Different capital letters show statistical differences within each treatment over time (columns) at the 5% level using Duncan’s *post-hoc* test. Different lower case letters show statistical differences between treatments at each time point (lines) at the 5% level.

**Table S2 Survival rates of *Aedes aegypti* larvae following exposure to different treatments under simulated field conditions**

| **SURVIVAL RATES (%)** | | | | |
| --- | --- | --- | --- | --- |
| **TIME (days)** | **F + N** | **F** | **N** | **CONTROLS** |
| 0 | 25.5 ± 8.8 Aa | 26.6 ± 7.9 Aa | 32.2 ± 8.5 Ab | 80 ± 2.6 Ac |
| 5 | 47.7 ± 7.3 Ba | 50 ± 5.2 Ba | 48.8 ± 7.6 Ba | 82.2 ± 2.6 BCb |
| 10 | 71.1 ± 5.6 Ca | 72.2 ± 4.3 Ca | 84.4 ± 2.9 Cb | 86.6 ± 2.4 Cb |

**F:** *Metarhizium anisopliae* conidial suspensions (1x10^9^ conidia mL^-1^)

**N**: Neem oil 0.1%

The different suspensions/solutions were all prepared at the same time and larvae added to the pots at 5-day intervals. Time zero: larvae added to freshly prepared suspensions/solutions.

Different capital letters show statistical differences within each treatment over time (columns) at the 5% level using Duncan’s *post-hoc* test. Different lower case letters show statistical differences between treatments at each time point (lines) at the 5% level.

**Table S3** Germination rates (mean % ± SD) of *Metarhizium anisopliae* conidia following exposure to UV-B when formulated in two concentration of neem oil or Tween

| **UV-B Exposure times** | **Treatment F+N (1%)** | **Treatment F+N (0.01%)** | **Treatment F** |
| --- | --- | --- | --- |
| 30 min | 82.9 ± 0.47 Aa | 82.1 ± 1.24 Aa | 82.7 ± 1.24 Aa |
| 1 h | 82.1 ± 1.69 Aa | 61.8 ± 0.94 Bb | 48.2 ± 0.94 Bc |
| 2 h | 80.3 ± 1.69 Aa | 46.2 ± 0.47 Cb | 32.8 ± 1.41 Cc |
| 3 h | 68.1 ± 1.41 Ba | 32.4 ± 1.69 Db | 19.7 ± 0.81 Dc |
| 4 h | 38.3 ± 1.24 Ca | 20.5 ± 0.94 Eb | 9.16 ± 0.47 Ec |
| 5 h | 16.8 ± 0.94 Da | 9.9 ± 0.47 Fb | 1.02 ± 0.94 Fc |

F: Fungus

N: Neem oil at a final concentration of 0.01 or 1%.

The results were analyzed using a one-way ANOVA and Duncan’s post-hoc at the 5% signifiance level.

Differences in germination rates for each treatment over time (different capital letters indicate significant differences between germination rates over time), whilst differences between treatments for each time point were also compared (different small letters indicated significant differences between treatments at specific UV exposure times).

The control treatments (no exposure to UV-B) are not shown in this table. Conidia were suspended in either N (1%), N (0.01%) or tween only (F). The germination rates were not significantly different (P>0.01). The germination rates were **F+N (1%)** 81.7% (± 0.47); **F+N (0.01%)** 82.5% (± 0.47) and **F** 83.1% (± 1.24).

**F values: comparing each treatment over time**

**F+N 1% 30 min to 5 h:** F_5,17_ = 46.07; P< 0.01

**F+N 0,01% 30 min to 5 h:** F_5,17_ = 9270.3; P< 0.01

**F: 30 min to 5 h:** F_5,17_ = 94580; P< 001

**F values: comparing each time point between treatments**

30 min P>0.01

1 h: F_2,8_ = 11724; P<0.01

2 h: F_2,8_ = 7761.3; P<0.01

3 h: F_2,8_ = 12.9; P<0.01

4 h: F_2,8_ = 4508.6; P<0.01

5 h: F_2,8_ = 314.7; P<0.01
